# Shining star-like appearance in Pott’s disease

**DOI:** 10.1590/0037-8682-0097-2025

**Published:** 2025-06-16

**Authors:** Li-jie Guo, Yong-Sheng Yu, Yi Zhang

**Affiliations:** 1 Department of Infectious Diseases, Shanghai Sixth People’s Hospital Affiliated to Shanghai Jiao Tong University School of Medicine, Shanghai, China.

A 31-year-old woman presented with a 12 -month history of increasing lower back pain and a limited range of motion. Physical examination revealed percussion pain in the L1 and L2 vertebrae. The white blood cell count was 6.1×10^9^/L, with 65.3% neutrophils and 20.2% lymphocytes. Inflammatory marker levels were elevated, with an erythrocyte sedimentation rate of 79 mm/h and C-reactive protein level of 46 mg/L. Computed tomography (CT) scan revealed apparent destruction of the L1 and L2 vertebrae, severe collapse of the L2 vertebra, obvious kyphosis (type B, according to the extent of initial vertebral loss^1^ ) with involvement of the L1 and L2 vertebrae (Figure 1), and apparent bony fragments scattered among the L2 vertebral body resembling “shining stars” (Figure 2). Magnetic resonance imaging demonstrated apparent destruction of the L1 and L2 vertebrae, accompanied by paravertebral abscesses (Figure 3A) and obvious kyphosis resulting from significant destruction of the contiguous vertebral segments (L1 and L2 vertebrae) (Figure 3B). Subsequently, the patient underwent surgery. *Mycobacterium tuberculosis* was cultured from the pus. The patient was diagnosed with tuberculous spondylitis (TS) and received anti-tuberculosis therapy for 12 months. She responded satisfactorily the medical treatment.

**FIGURE 1:**
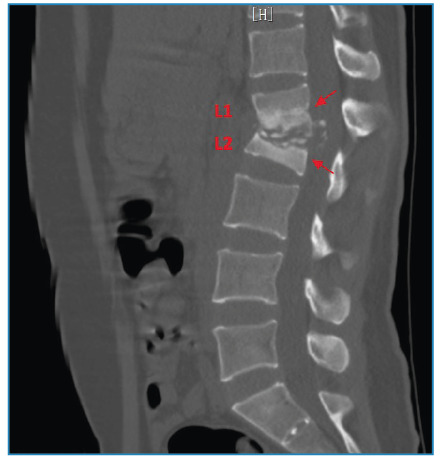
Computed tomography scan (sagittal section) showing apparent destruction of the L1 and L2 vertebrae, severe collapse of the L1 vertebra, and obvious kyphosis (type B) with involvement of the L1 and L2 vertebrae (red arrows).

**FIGURE 2:**
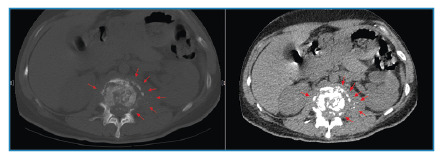
Computed tomography scan (transverse section) showing apparent bony fragments scattered among the L2 vertebral body resembling “shining stars” (red arrows).

**FIGURE 3:**
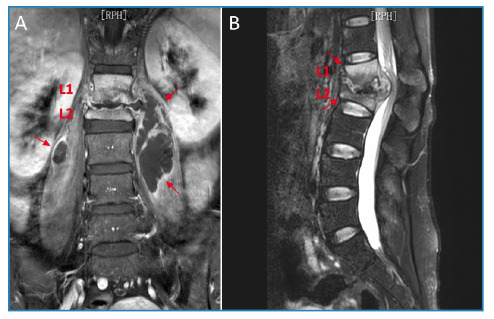
**A:** Magnetic resonance image (coronal section) showing apparent destruction of the L1 and L2 vertebrae accompanied by paravertebral abscesses (red arrows). **B:** Magnetic resonance image (sagittal section) showing obvious kyphosis resulting from significant destruction of the contiguous vertebral segments (L1 and L2 vertebrae) (red arrows).

TS, known as ‘‘Pott’s disease”, is the most common cause of kyphotic deformity in many parts of the world^1^. The fragmentary type, which has an appearance of shining stars, is the most common pattern of vertebral destruction observed on CT scans^2^ ^-^ ^4^. The appearance is a typical radiological feature that further helps distinguish TS from other infectious diseases of the spine^4^.

## References

[1] Rajasekaran S (2013). Natural history of Pott’s kyphosis. Eur Spine J.

[2] Rivas-Garcia A, Sarria-Estrada S, Torrents-Odin C, Casas-Gomila L, Franquet E (2013). Imaging findings of Pott’s disease. Eur Spine J.

[3] Sinan T, Al-Khawari H, Ismail M, Ben-Nakhi A, Sheikh M (2004). Spinal tuberculosis: CT and MRI feature. Ann Saudi Med.

[4] Ding HQ, Yuan WQ (2021). Differential diagnosis and treatment of brucella spondylitis and spinal tuberculosis (Chinese). Chin J Orthop.

